# Mismatch Between Specific and Genetic Diversity in an Evergreen Broadleaf Forest in Southeast China: A Study Case of 10.24 ha Forest Dynamics Plot of Huangshan

**DOI:** 10.3389/fpls.2021.706006

**Published:** 2022-01-31

**Authors:** Lei Xie, ShuiFei Chen, YueYao Feng, Yao Li, Lu Wang, LiHeng He, LiQun Huang, Jun Wu, Ke Guo, Hui Ding, YanMing Fang

**Affiliations:** ^1^Key Laboratory of State Forestry and Grassland Administration on Subtropical Forest Biodiversity Conservation, Co-Innovation Center for Sustainable Forestry in Southern China, College of Biology and the Environment, Nanjing Forestry University, Nanjing, China; ^2^State Environmental Protection Key Laboratory on Biosafety, State Environmental Protection Scientific Observation and Research Station for Ecological Environment of Wuyi Mountains, Biodiversity Comprehensive Observation Station for Wuyi Mountains, Nanjing Institute of Environmental Sciences, Ministry of Ecology and Environment, Research Center for Nature Conservation and Biodiversity, Nanjing, China; ^3^School of Civil Engineering, Nanjing Forestry University, Nanjing, China; ^4^Bureau of Parks and Woods of Huangshan Management Committee, Huangshan, China

**Keywords:** SGDC, biodiversity, soil properties, topography, community, population, *Castanopsis eyrei*

## Abstract

For a long time, forestry management has often focused on the protection of species diversity, and mistakenly believed that protecting species diversity protects genetic diversity. Therefore, research that integrates community ecology and population genetics has become important because it can help elucidate whether the targets for protecting specific and genetic diversity are congruent. In this study, we have emphasized the impact of the community on the population because no previous studies have considered the community composition of a place *a priori*. Based on the Huangshan 10.24 ha dynamics forest plot, we *a priori* considered the community composition in the plot to test species-genetic diversity among the tree layers. Firstly, a redundancy analysis (RDA) found that *Castanopsis eyrei* and *Pinus massoniana* were the dominant species. Secondly, specific and genetic diversity are not congruent in Huang Shan. Finally, the structural equation model (SEM) showed that the different degrees of response by community composition and population structure to environmental heterogeneity are the main reasons for the mismatch between species diversity and genetic diversity. The results suggest that we must focus on genetic diversity, as well as on protecting species diversity.

## Introduction

Biodiversity is fundamentally altered by some human activities such as overuse of land, hunting and CO_2_ emissions, and these processes have led to a continuous decline in biodiversity (Blowes et al., 2019). To date, the main method used to delay the decline in biodiversity is to establish nature reserves in threatened forest ecosystems (Yang et al., 2019a), but in the future, the red list of ecosystems should be adopted when designating protected areas where biodiversity processes and higher-level components can be maintained (Chen et al., 2020). As the two basic components of biodiversity, species diversity (SD) represents diversity across species, and genetic diversity (GD) represents genotype variation at the intraspecies level and its distribution patterns (Vellend et al., 2014a). Generally, mutation, selection, migration, and drift directly affect genetic variation and population genetic structure changes, and indirectly affect genetic diversity (Ellegren and Galtier, 2016; Zhang et al., 2018; Li et al., 2019). Environmental heterogeneity directly influences the processes and functions of the ecosystem, and indirectly influences the population structure and community composition by affecting plant physiology, biochemistry, morphology, and behavior (Suding et al., 2008). The protection of SD has been researched for a long time under the misunderstanding that to protect SD is to protect GD (Taberlet et al., 2012).

However, an increasing number of reviewers have investigated the relationships between these two levels of biodiversity in order to determine how the two levels influence each other (Vellend, 2003, 2005; Vellend and Geber, 2005). Since the first integrated research was published (Vellend, 2003), it has been accepted that there is a correlation between species and genetic diversity (SGDC). Based on the review (Antonovics, 1976), it has been suggested that the factors affecting SD and GD are similar, and it has been hypothesized that both SD and GD are responses to parallel environmental heterogeneity effects, such as area, resource availability, and disturbances (Vellend, 2003, 2004, 2005). Then, Vellend and Geber (2005) proposed three modes of interaction among environments, communities, and populations, and gave the mechanisms behind them. Briefly, a positive relationship would be found when ecological processes act on two levels. On the other hand, if there was an increase in the GD of a dominant species, then the species would increase their biomass, occupy a larger niche, and exclude other species, which would change community assembly. Furthermore, community also has an influence on GD by controlling the population size (Vellend and Geber, 2005).

Over the past 20 years, in order to verify the hypothesis proposed by Vellend and Geber (2005), a series of empirical studies were undertaken and three results emerged. A number of studies confirmed the positive SGDCs (Vellend, 2004; Wei and Jiang, 2012; Lamy et al., 2013; Laroche et al., 2015; Pfeiffer et al., 2018) because of parallel effects, such as connectivity, area, and interference etc., on two levels. However, under the interference of some natural or artificial factors such as habitat fragmentation characteristics, artificial fertilization, roadworks, and soil phosphorus limit, SD and GD might respond differently. In this case, there was a negative or irrelevant relationship between SD and GD (Puşcaş et al., 2008; Silvertown et al., 2009; Taberlet et al., 2012; Xu et al., 2016; Bertin et al., 2017; Wei et al., 2018). Therefore, it is not clear whether there is a correlation between SD and GD. At present, there is no unanimous conclusion on SGDCs and there have been few empirical studies on SGDCs, especially in China (Wei and Jiang, 2012; Xu et al., 2016; Wei et al., 2018). Furthermore, the differences among the existing study species, population structures, community assemblies, scales, indexes used to measure SGDCs, and the experimental designs might have led to contrary conclusions (Vellend et al., 2014b; Whitlock, 2014).

Some literature has already emphasized the impact of community on the population (Vellend et al., 2014b; Lamy et al., 2017; Xie et al., 2021). However, when we examined SGDCs from the viewpoint of empirical studies, we found that they did not consider the community composition of a place, *a priori*. For example, most previous investigations that used randomly sampled layout plots tended to conduct studies on herbs with a short lifecycle (Wei and Jiang, 2012; Pfeiffer et al., 2018; Reisch and Schmid, 2018). The Huangshan Forest Dynamics Plot is a crosscutting research platform for biodiversity science to figure out species distribution and composition after census which can be help to understand the local community composition in advance. Based on the existing species distribution, we can select representative subplots to carry out SGDC research.

In this study, our primary aims were to determine the correlation between SD and GD in the tree community and to test the hypothesis that GD within population and SD within community co-vary in space (Antonovics, 1976, 1992, 2003; Vellend, 2005; Vellend et al., 2014a). Three alpha diversity indices of species were selected to evaluate SD and several genetic parameters were used to estimate GD. Topography and soil properties were used to test the SD and GD responses to environment heterogeneity to better understand whether there were parallel effects on SD and GD.

This study specifically addressed the following fundamental questions: (1) Is there a positive correlation between SD and GD and (2) are the responses of community and population to environmental heterogeneity consistent? If they are not consistent, then how are the population and community affected by different environmental heterogeneities?

## Materials and Methods

### Study Site

**FIGURE 1:**
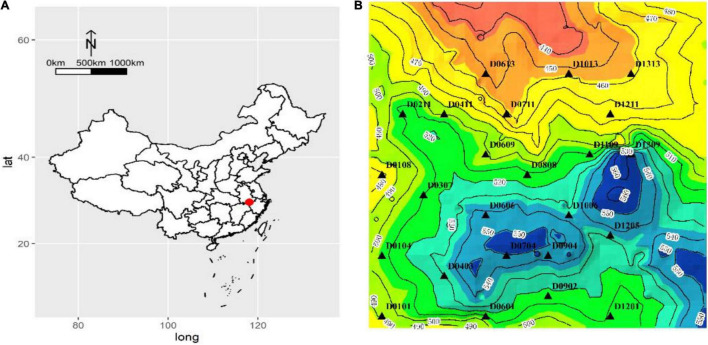
**(A)** Geographic location of the Forest Dynamics Plot at Huangshan (HS) and **(B)** the sampling points (triangles).

Huangshan (HS) is a member of World Biosphere Reserve, Cultural Double Heritage, and Priority Areas for Biodiversity Conservation. This study was conducted in the Forest Dynamics Plot (10.24 ha) at HS, a subtropical evergreen broadleaved forest, Anhui Province, Southeast China, with the geographical coordinates 30°8′26″N, 118°6′38″E and an elevation of 400–600 m (Figure 1; Ding et al., 2016). The annual average temperature is 7.8°C, the average temperature in the hottest month (July) is 17.7°C, and the average temperature in the coldest month (January) is 3.1°C. The average annual rainfall and duration of the rainy season are 2,394.5 mm and 182 days, respectively (Ding et al., 2016). The forest dynamics plot was established in the summer of 2014 in western Huangshan, and a re-census was carried out in 2019. The forest dynamics plot is one of the important biodiversity observation platforms for subtropical evergreen broad-leaved forests in China. The leaf samples for this study were collected in August 2020.

### Experimental Design

We firstly selected *Castanopsis eyrei* as the focal species. It is a large, evergreen, subtropical tree that grows in broadleaf forests in southeastern China and is also a dominant species at HS (Xu et al., 2019; Staab et al., 2021). In addition, *C. eyrei* is also the species with the highest importance value in HS forestry dynamics plot (Ding et al., 2016).

Here, we measured SGDCs under different community structures based on species diversity indices in 256 plots. The 10.24 ha forest dynamics plot was divided into 256 subplots that were 20 × 20 m in size. The community assembly differences among subplots were assessed using the following methods. We first calculated three species diversity indices (the Shannon-Wiener index, the Gini-Simpson index, and the Pielou index) based on the 2019 re-census data. Based on the subplot species diversity indices, we then used a principal components analysis (PCA) to divide the 256 subplots into four groups (Supplementary Figure 1). Group I had a negative Pielou index value, Group II had positive Simpson index and Shannon-Wiener index values, Group III had a positive Pielou index value, and Group IV had negative Simpson index and Shannon-Wiener index values (PC_1_ + PC_2_ = 97.78%). Secondly, we considered the numbers of focal species (>5) in each plot and the distance (>20 m) among the subplots. After considering these factors, we randomly selected 24 subplots from the four groups among the 256 subplots at HS.

Each selected subplot was considered as a population of the focal species based on a previous study (Xu et al., 2016). Here, we considered the distance among the focal species in the subplots to eliminate any possibility that they might have come from the same plant material. Therefore, we did not choose all focal species in the selected plots. Each 20 × 20 m subplot was also divided into 16 5 × 5 m grids and 0–3 individuals were selected from these grids. A total of 601 trees of the focal species in 24 subplots were selected (Supplementary Table 1).

### Measuring Topography and Soil Properties

Three topographic variables were quantified for each 20 × 20 m subplot. These were mean elevation, slope, and convexity, and these variables were calculated from the elevation data. In each plot, the mean of four vertices was used as the elevation of the plot; the average of the angles between the plane formed by any three angles of the target plot and the horizontal plane was taken as the slope of the plot; and the elevation of the target plot minus the average elevation of the surrounding eight squares was used as the convexity of the plot (Liu et al., 2013). Nitrogen (N) and phosphorus (P) are generally considered to be the two most restricted elements in terrestrial vegetation, and they play a vital role in the composition of plant communities. Carbon (C) and potassium (K) are important nutrients for plants. pH is believed to change the physical and chemical properties of soil and affect plant growth. The soil sampling methods followed the procedures described in Sun et al. (2020), and a total of 72 soil samples were obtained from HS. In short, one sampling point at the center of the subplot, and the other four points at the middle point between the center and the four corners, respectively. After removing the litter on the surface of the soil, we used a soil auger to sample at depth of 0–15 cm. Five soil traits were determined for each sample. Soil organic carbon (SOC) was measured using the K dichromate volumetric method; total nitrogen (TN) in the soil was determined using elemental analysis (MD SpectraMax 190, United States); total phosphate (TP) was assessed by molybdenum-antimony colorimetry; and total potassium (TK) was analyzed by a flame photometer. The soil pH was determined using a glass electrode at a 1:2.5 soil/water ratio (Pansu and Gautheyrou, 2006). Each soil property was measured three times. For the first time, we determined the impact and importance of various environmental factors on SD and GD in the HS metacommunity.

### Measuring Species Diversity

A total of four species diversity indices [Tree richness (S), the Shannon-Wiener index (SD_SW), the Gini-Simpson index (SD_SI), and the Pielou index (SD_J)] were calculated by the “vegan” package (Oksanen et al., 2017). All indices were based on the 2019 re-census data. Four indices were calculated as follows (Magurran, 1988):

Tree richness:


(1) S=n

Where S is the species richness in each 400 m^2^ subplot, and *n* is the number of tree species with a diameter at breast height ≥ 1 cm.

Shannon-Wiener index:


(2) $$H=-\sum_{i=1}^{S}{}_{p_{i}\log_{x}p_{i}}$$

Where *p*_*i*_ is the relative abundance of species *i*, *p*_*i*_ = *N*_*i*_/*N*_0_, *N*_*i*_ is the abundance of the *i* species, and *N*_0_ is the sum of the abundances of all species.

Gini-Simpson index:


(3) $$GS=1-\sum_{i=1}^{S}p_{i}^{2}$$

Where *p*_*i*_ is the relative abundance of species *i*, *p*_*i*_ = *N*_*i*_/*N*_0_.

Pielou index:


(4) $$J=\frac{H}{\log_{x}S}$$

Where *H* is the Shannon-Wiener index, *S* is the tree richness.

### Measuring Genetic Diversity

To our knowledge, few studies have been conducted to detect SGDC based on a biodiversity dynamics observation plot, and most previous studies used amplified fragment length polymorphism (AFLP) to detect a population structure (Xu et al., 2017). In this study, we combined the multiplexed amplification of SSRs with high throughput sequencing to obtain more genotyping (Yang et al., 2019b; Lepais et al., 2020). Compared with traditional methods that rely solely on fragment size for genotyping, using next-generation sequencing technology for SSR genotyping can obtain higher levels of genetic polymorphism results (Sarhanova et al., 2018).

Leaf samples belonging to *C. eyrei* were taken from the selected 24 subplots and 601 samples were immediately dried in a plastic bag containing silica gel. Ten to 15 leaves were taken from each individual. We tested 69 nuclear microsatellite loci developed for Fagaceae, from which 30 (Supplementary Table 2) were selected as they had suitable levels of polymorphisms. DNA was extracted from silica-dried leaves using a DNAeasy Plant Mini Kit (Tiangen, DP350). Primers were designed and synthesized, and their DNA quality was evaluated. Then, PCR amplification was performed for each SSR locus. Optimal primers with clear bands were selected for further multiplex PCRs. Up to 30 loci primers were pooled together and adjusted using the results to achieve equal amounts of the products for each locus, to optimize primer compatibility, and to avoid undesired primer pairing. All the PCR products were pooled prior to library preparation and sequenced on the Illumina HiSeq2500 platform (PE150 bp) by Genesky Biotechnologies, Inc. (Shanghai, China). The raw reads were analyzed by pipeline, and quality control was conducted using FastQC, read merging in FLASH, and by the construction of reference alignments using Blastn. The number of SSR alleles was calculated using aligned reads and the sequence data. The SSR motif and repeat number were listed after two step adjustments, including slippage adjustment and amplification efficiency adjustment. Data analyses were based on SSR genotyping results, GenoDive version 2.0b27 (Meirmans and Van Tienderen, 2004) was used to calculate the observed number of alleles Na, effective number of alleles Ne, Shannon’s information index I, observed heterozygosity Ho, expected heterozygosity He.

### Data Analysis

All the statistical analyses were conducted using R 4.0.3 (R Development Core Team, 2020). The correlation between species diversity and genetic diversity was determined by calculating Pearson correlation coefficients. The Chi-squared Test was used to test whether the variables were significantly correlated. The effects of soil and topography on the SD and GD were investigated using hierarchical partitioning to obtain the explanatory rate of each explanatory variable (Lai and Pedro, 2020). In the hierarchical partitioning process, pH, SOC, TN, TP, and TK were set to one group and mean elevation, slope and convexity were set to another group variables. A redundancy analysis (RDA) was employed across subplots from different groups to evaluate the relationships among the soil properties, topography, and plant community structures using the “vegan” package (Oksanen et al., 2017). Five soil properties (pH, SOC, TN, TP, and TK) and three topographic variables (slope, mean elevation, and convexity) were included in the RDA. In the RDA, we first determined the Hellinger value for plant richness to eliminate the influence of extreme values and reduce the weight of highly abundant species. The Ezekiel (1930) method was used to obtain the adjusted *R*^2^. A permutation test (*n* = 999) was used to test the significance of the RDA and the constrained ordination axes in the reduced model (Borcard et al., 2018).

The effects of topography and soil properties on SD and GD were examined using the following model:


(5) Y=b0+bX1+1bX2+2bX3+3…+ε

where Y is the index for SD or GD; X_1_, X_2_, X_3_ represent the variables of soil and topography; and ε is the random sampling error. Linear analysis with a restricted maximum likelihood was conducted to measure the relationship between soil, topography, species diversity, and genetic diversity. Before we ran the model, we conducted data conversion on response variables Y so that the model residuals met the conditions for normality. The variables SD_J, SD_SI, SD_SW, I, and Ne were SD_J/1-SD_J, SD_SI/1-SD_SI, square, quadrillion, and cubed transformed to ensure that the residuals remained normal. All model residuals were checked for normality by the “performance” package (Nakagawa et al., 2017).

The “vegan” package (Oksanen et al., 2017) was used to conduct a PCA to condense the topography and soil properties into a small number of uncorrelated variables for further path diagrams. The influence of topography and soil properties on species diversity and genetic diversity was determined using path analyses conducted based on local estimation by the “piecewise SEM” package (Lefcheck, 2016). We first ran a meta model (Supplementary Figure 1) and Shipley’s test of directed separation (*d*-separation test) was used to find the missing path. After adding the significant path, we then generated the final model to determine relationships among the community assembly, population of the *C. eyrei* and environmental heterogeneity.

## Results

### Species Diversity and Genetic Diversity

A total of 87 species with a diameter at 1.3 m height (DBH) ≥ 1 cm were observed in 24 subplots (Supplementary Table 3). We found that the index of different species diversity has a large range of variation. Species richness, the Pielou index (SD_J), the Shannon-Wiener index (SD_SW), and the Simpson index (SD_SI) values for SD were from 10 to 36, 0.610 to 0.829, 1.496 to 2.890, and 0.639 to 0.921, respectively (Supplementary Table 4). Apart from *C. eyrei*, the most frequent species were *Pinus massoniana*, *Vaccinium mandarinorum*, *Symplocos setchuensis*, and *Rhododendron simsii* (Supplementary Table 3).

A total of 1,438 alleles at 30 loci were revealed across 601 individuals from 24 *C. eyrei* populations. The observed number of alleles (Na) among subplots varied from 3.395 to 8.026; the effective number of alleles (Ne) among subplots varied from 2.648 to 4.189; the Shannon’s information index (I) among subplots varied from 0.987 to 1.451; the observed heterozygosity (Ho) among subplots varied from 0.517 to 0.568; and expected heterozygosity (He) among subplots varied from 0.547 to 0.644 (Supplementary Table 4).

### Topography and Soil Properties

The elevation and slope of the 24 subplots ranged from 442.46 to 548.62 m and from 7.79 to 47.04°, respectively (Supplementary Table 1). The convexity of the subplots was also highly variable. The means for each soil property were 72.7 ± 27.6 g/kg (SOC), 3.78 ± 0.801 g/kg (TN), 0.335 ± 0.06 g/kg (TP), 21.8 ± 1.69 g/kg (TK), 4.90 ± 0.21 (pH), 19.2 ± 5.32 (C: N) (Supplementary Table 6).

### Relationships Between Species Diversity and Genetic Diversity, and Environment Heterogeneity

**FIGURE 2:**
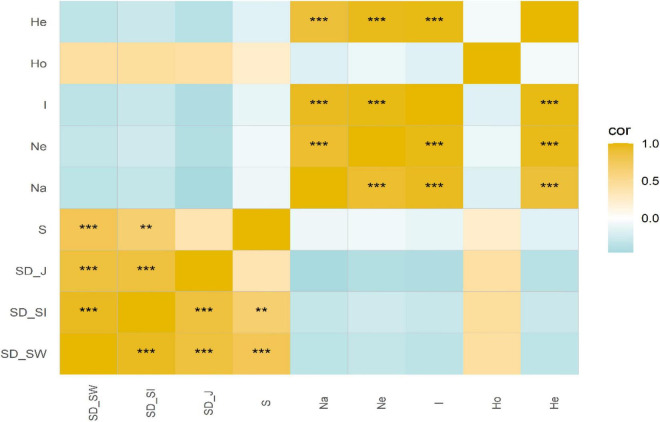
Correlations between species diversity and genetic diversity. **P < 0.01, ***P < 0.001.

**FIGURE 3:**
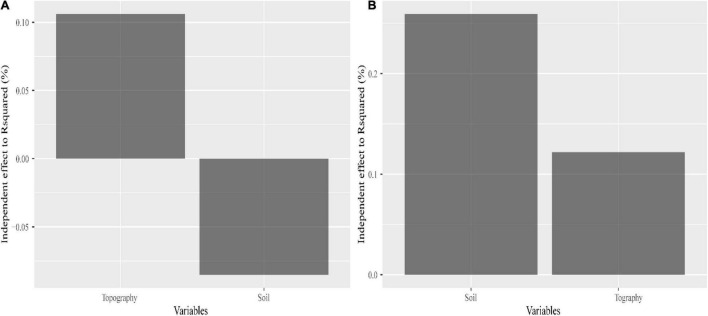
Contributions made by a single explanatory variable to species diversity **(A)** and genetic diversity **(B)**.

There was no significant correlation between SD and GD for *C. eyrei* (Figure 2). However, the internal indices within SD and GD were significantly correlated. We found that both topography and soil had significant effects on genetic diversity (Figure 3B and Table 1). However, soil had no effect on community composition (Figure 3A and Table 1).

**TABLE 1:** Effects of soil and topography on the SD and GD indices for species diversity (SD) and genetic diversity (GD) in the subplots (S, species richness; SD_SI, Simpson index; SD_J, Pielou index; SD_SW, Shannon-Wiener index; Na, observed number of alleles; Ne, effective number of alleles; I, Shannon’s information index; Ho, observed heterozygosity; He, expected heterozygosity).

Source	S	SD_SI	SD_J	SD_SW	Na	Ne	I	Ho	He
**ELE**	−0.14*	−0.005*	–0.005	–0.02	0.004*	0.04	0.005	−	−
**CON**	0.1	–0.002	−	0.02	0.002*	0.21	0.02	−	−0.0005*
**SLO**	0.03	0.002	−	0.004	0.001	0.12	0.009	−	−
**SOC**	–0.003	−0.02*	–0.02	–0.02	–0.001	–0.08	–0.005	−	−
**TN**	4.69	0.19	0.19	0.84	0.6	10.57*	0.78	−	0.01
**TP**	–39.85	8.55	8.55	3.15	−0.13*	−166.33*	−15.72*	0.008	−0.18*
**TK**	1.59	–0.03	–0.03	0.18	–0.04	–0.539	–0.06	0.004	–0.003
**PH**	–1.23	–1.77	–1.76	–2.03	–0.9	–15.00	–1.39	–0.02	–0.02

*P < 0.05, −, the value is too small to be placed in the table.

**FIGURE 4:**
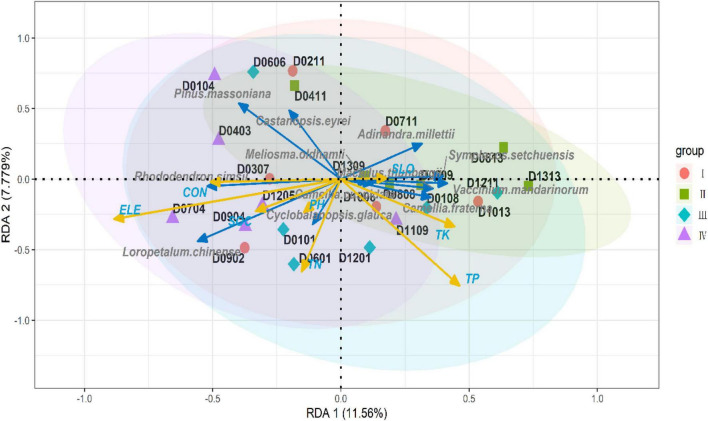
Redundancy analysis (RDA) ordination plot for the multivariate effects of soil properties and topography. The blue arrow is the response variable and the yellow arrow is the explanatory variable. The shaded area indicates the confidence interval for each component (ELE, elevation; CON, convexity; SLO, slope).

After adjustment, R squared was 0.2954, and axes 1 and 2 explained 11.56 and 7.78% of the overall variance in the plant communities, respectively (both whole model and each canonical axis, *p* < 0.05). *Castanopsis eyrei* and *P. massoniana* were almost negatively correlated with all soil properties and richness of other dominant species; however, both species were positively correlated with topography, indicating that dominant species, such as *C. eyrei* and *P. massoniana*, were significantly influenced by environmental heterogeneity (Figure 4).

**FIGURE 5:**
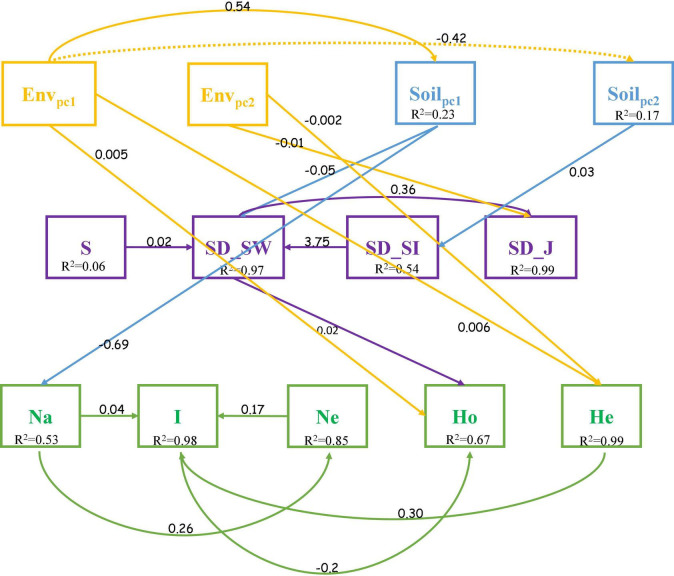
Path diagrams for analyzing the relationship between topography, soil properties, community assembly, and genetic structure (Fisher C = 46.355, *P*-value = 0.902). Env_*pc1*_, PC_1_ for topography; Env_*pc2*_, PC_2_ for topography; Soil_*pc1*_, PC_1_ for Soil; Soil_*pc2*_, PC_2_ for Soil.

**FIGURE 6:**
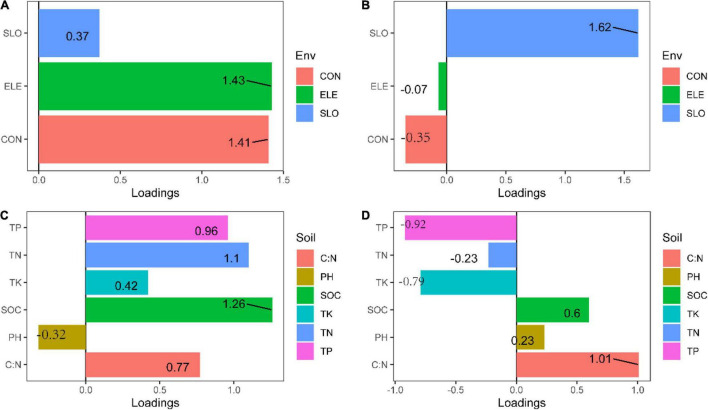
Loadings for the topography and soil properties principal components (PCA) **(A)** PC_1_ for topography, **(B)** PC_2_ for topography, **(C)** PC_1_ for soil properties, and **(D)** PC_2_ for soil properties.

A cross structural equation modeling (SEM) analysis was used to explore the relationship between topography, soil properties, community composition, and the genetic structure of *C. eyrei*. The best fit model showed that topography had an effect on both community and population. However, soil properties only influenced the observed number of alleles. A slight relationship between SD and GD was found by the SEM, and the Shannon-Wiener index (SD_SW) for community showed there was a positive relationship with observed heterozygosity (Ho) (*p* < 0.01, Figures 5, 6).

## Discussion

This study differed from some previous studies because they reported a positive correlation between SD and GD (He et al., 2008; Lamy et al., 2013; Pfeiffer et al., 2018). Our study revealed that population structure was not congruent with community assembly in a plant community (Figure 2). Similar to previous studies (Taberlet et al., 2012), our results suggested that SD cannot be simply substituted by GD in a subtropical forest, at least in HS. Furthermore, based on a large sample size and 30 loci, we showed that topography and soil properties had different influences on community assembly and genetic structure. Environmental heterogeneity has significant effects on genetic structure, but has no marked impacts on community assembly. Below, we discuss the observed patterns for the responses of two levels of biodiversity to environmental heterogeneity and the potential mechanisms behind the correlation between the SD and GD.

### Impacts of Environmental Heterogeneity on Community and Genetic Structure

The SGDCs improve our understanding of the relationships between the intraspecific variation and species diversity, which can have an influence on the community assembly (Lamy et al., 2017). Before we discuss the mechanisms behind the correlation between the species diversity and genetic diversity, we should understand how the *C. eyrei* population and HS community respond to environmental heterogeneity.

We discovered that both soil properties and topography had effects on population structure of *C. eyrei* (Figure 3). Based on the linear regression, we further found that phosphorus plays a key role in the growth of *C. eyrei* population (Table 1). We also found that elevation is negatively correlated with phosphorus (Figure 5), and the richness of other species decreases, which means that higher elevation are more suitable for *C. eyrei* growth and they may become the top succession species in the area. Environmental filter theories predict that plant diversity changes along the environmental gradient, and this can be interpreted as the environment filtering out of some plants that are not suited to the local environmental conditions (Laliberte et al., 2014; Blanchard et al., 2020). This discovery is meaningful, and suggests that *C. eyrei* is the main constructive species in the Huangshan area. Therefore, we chose *C. eyrei* as the focal species. However, we found that the community only responds to soil nutrients (Figure 3), which means topography is the main factor shaping community assembly in HS.

### Mechanisms Behind the Correlation Between the Species Diversity and Genetic Diversity

It is theoretically believed that the parallel impact of SD and GD on the community and the population would lead to a positive SGDC (Vellend, 2005). However, in this study, no significant correlation between the two was found in the forest dynamics plot (Figure 2). It is not the first time that a mismatch SD and GD has been recorded. In an integrated study of flora, Lamy et al. (2017) found that only 11% of the studies reported that SD and GD were significantly related. There have also been related previous studies on SGDC in one location. Most studies have reported non-significant or negative correlations (Avolio and Smith, 2013; Reisch and Schmid, 2018; Wei et al., 2018). When comparing our results to these older studies (Vellend, 2004; Lamy et al., 2013; Frey et al., 2016), it must be pointed out that connectivity and area will have effects on SGDC. Discrete and continuous sampling methods often determine whether we can find a positive SGDC. No significant correlation was found in a forest dynamics plot and grassland station (Avolio and Smith, 2013; Reisch and Schmid, 2018). This result has been used to successfully account for the island biogeography theory. A delineated vegetation community is generally regarded as an island-like research system (Reisch and Schmid, 2018). A small area often leads to increased drift, decreased internal connectivity, and loss of alleles, which leads to a decrease in genetic diversity and species diversity. Therefore, a weaker or insignificant SGDC correlation was often recorded.

In line with previous studies, the effects of environmental heterogeneity on species diversity and genetic diversity were different (Figure 3; Avolio and Smith, 2013). In this study, we found that the total phosphorus had a negative effect on the *C. eyrei* population, which showed that low phosphorus levels restrict the growth of *C. eyrei*. As a result, the effective population decreases, resulting in a decrease in genetic diversity. A further analysis by SEM showed that the community assembly response to soil nutrition was significantly smaller than that of a single population to soil nutrition (Figure 6). It is worth noting that the Shannon-Wiener index was positively related to observed heterozygosity (Figure 6). The results show that community assembly affected the *C. eyrei* population.

When comparing our results to those of older studies, it must be pointed out that the niche of the focal species, *C. eyrei*, was not congruent with other species and this might be the reason why there was no relationship between species diversity and genetic diversity. In this study, we found that *C. eyrei* and *P. massoniana* richness was negatively related to most dominant species. In addition, we also found that the population richness was negatively correlated with almost all soil nutrients (Figure 5). This may be due to the fact that *C. eyrei* competition for resources, such as phosphorus, is more specialized than other species in the community (Lamy et al., 2017). The increase in the availability of phosphorus might increase the average population size of the focal species and decrease all other species in the community. Therefore, there was a non-significant relationship between species diversity and genetic diversity.

## Conclusion

Our findings have significant implications for elucidating species coexistence and biodiversity maintenance in HS plant assembly studies, especially in evergreen broadleaf forest. It is difficult to consider the process of biodiversity and higher-level components in a species-by-species assessment in biodiversity protection. Here, we used community investigation, molecular biology combined with ecological modeling to figure out the community assembly rules in Huangshan. We found that there were no SGDCs in the plant community investigated by this study, demonstrating that the importance of the genetic diversity cannot be ignored when you only place emphasis on species diversity. Furthermore, our findings revealed that SD and GD respond to topography and soil properties differently, which means that species diversity is not congruent with genetic diversity. This research builds on our full understanding of the relationship between α-diversity of gene and species (α-SGDC). Next, we would develop the relationship between genetic and community dissimilarities (β-SGDC).

## Data Availability Statement

The original contributions presented in the study are included in the article/Supplementary Material, further inquiries can be directed to the corresponding author/s.

## Author Contributions

LX: idea of manuscript (lead), all data collection and analysis (lead), and text writing (lead). LW, LQH, JW, and KG: resources (equal) and data collection of species diversity (equal). SC and HD: resources (equal), data collection of species diversity (equal), and writing–review and editing (supporting). YYF and YL: data collection of species diversity (equal) and writing–review and editing (equal). LHH: data collection of topography (equal). YMF: resources (equal), supervision (lead), validation (equal), and writing–review and editing (support). All authors read and approved the final manuscript.

## Conflict of Interest

The authors declare that the research was conducted in the absence of any commercial or financial relationships that could be construed as a potential conflict of interest.

## Publisher’s Note

All claims expressed in this article are solely those of the authors and do not necessarily represent those of their affiliated organizations, or those of the publisher, the editors and the reviewers. Any product that may be evaluated in this article, or claim that may be made by its manufacturer, is not guaranteed or endorsed by the publisher.
