# Supplementary material for: Mismatch Between Specific and Genetic Diversity in an Evergreen Broadleaf Forest in Southeast China: A Study Case of 10.24 ha Forest Dynamics Plot of Huangshan
Source: Front Plant Sci. 2022 Jan 31;12:706006. doi: 10.3389/fpls.2021.706006 (PMC8841795; doi:10.3389/fpls.2021.706006)
Supplement: Supplementary file 2 [file Data_Sheet_2.docx]

Table S1 Sampling situation of each subplot

| Subplot | Elevation | Number1 | Number2 |
| --- | --- | --- | --- |
| D0101 | 496.62 | 31 | 22 |
| D0104 | 501.9 | 24 | 21 |
| D0108 | 488.7575 | 23 | 21 |
| D0211 | 499.13 | 253 | 49 |
| D0307 | 493.785 | 76 | 35 |
| D0403 | 530.86 | 88 | 34 |
| D0411 | 481.8125 | 164 | 39 |
| D0601 | 535.2825 | 25 | 21 |
| D0606 | 528.5475 | 184 | 47 |
| D0609 | 493.9675 | 46 | 24 |
| D0613 | 446.21 | 26 | 17 |
| D0704 | 548.025 | 36 | 24 |
| D0711 | 455.8975 | 38 | 26 |
| D0808 | 495.6975 | 40 | 26 |
| D0902 | 530.5125 | 25 | 18 |
| D0904 | 548.4075 | 35 | 25 |
| D1006 | 530.58 | 16 | 15 |
| D1013 | 442.4575 | 30 | 25 |
| D1109 | 490.87 | 33 | 23 |
| D1201 | 512.5825 | 22 | 21 |
| D1205 | 548.6175 | 60 | 38 |
| D1211 | 464.58 | 15 | 9 |
| D1309 | 494.1875 | 67 | 18 |
| D1313 | 456.415 | 5 | 3 |

Number1: The actual number of *Castanopsis eyrei* in the subplot; Number2: The number of *C. eyrei* samples in the subplot

Table S2Characteristics of thirty SSR primers studied

| **ID** | **Repeat sequence** | **Size range (bp)** | **Reference** |
| --- | --- | --- | --- |
| Ccu62F15 | (TC)_17_ | 141-163 | Ueno et al. 2003 |
| Ccu90T17 | (TC)_23_ | 156-190 | Ueno et al. 2003 |
| Ccu87F23 | (TC)_19_ | 267-289 | Ueno et al. 2003 |
| Cch10 | (GTTTTG)_3_(GT)_8_ | 184-199 | Huang et al. 2009 |
| Cch12 | CAAC(CA)_2_GAAC | 150-175 | Huang et al. 2009 |
| Cch14 | (CACCCA)_5_ | 153-157 | Huang et al. 2009 |
| Ccu16H15 | (TC)_16_ | 136 | Shi et al. 2011a |
| Ccu28H18 | (CT)_26_ | 140 | Shi et al. 2011a |
| Ccu33H25 | (TG)_11_(TC)_15_ | 197 | Shi et al. 2011a |
| Ch2 KF725651 | (AC)6(AG)14 | 144-178 | Jiang et al. 2015 |
| Ch4 KF725653 | (AC)6(AG)6 | 213-281 | Jiang et al. 2015 |
| Ch5 KF725654 | (AC)6(AG)8 | 166-198 | Jiang et al. 2015 |
| Ch9 KF725658 | (AC)6(AG)5 | 167-195 | Jiang et al. 2015 |
| CS43 | (CT)9 | 92-106 | Shi et al. 2011b |
| CS92 | (GA)12…(AT)3 | 151-171 | Shi et al. 2011b |
| CS561 | (CT)20 | 316-352 | Shi et al. 2011b |
| CS620 | (CT)12 | 118-154 | Shi et al. 2011b |
| CS627 | (CT)16 | 190-232 | Shi et al. 2011b |
| CS687 | (CT)12 | 129-151 | Shi et al. 2011b |
| CFA04 | (AAG)11 | 174-202 | Shi et al. 2011b |
| CFA12 | (AGA)11 | 232-246 | Fu et al. 2010 |
| CFA25 | (GTT)7 | 224-240 | Fu et al. 2010 |
| CFA26 | (AAC)7 | 206-230 | Fu et al. 2010 |
| CFA31 | (GA)19 | 184-200 | Fu et al. 2010 |
| CFA35 | (AG)18 | 208-226 | Fu et al. 2010 |
| CFA45 | (AG)15 | 236-246 | Fu et al. 2010 |
| CFA46 | (TC)17 | 234-262 | Fu et al. 2010 |
| CFA57 | (TCT)23 | 174-202 | Fu et al. 2010 |
| CFA61 | (AC)11 | 180-200 | Fu et al. 2010 |
| CFA63 | (AT)7(TC)9 | 246-262 | Fu et al. 2010 |
| CFA71 | (AC)9…(AG)6 | 180-190 | Fu et al. 2010 |

Appendix 2 Table S3 Species information in 24 subplots

Table S4 Plots species diversity (SD_SW, Shannon-Wiener index; SD_SI, Simpson index; Pielou index, SD_J; S, species richness) and genetic diversity (Na, observed number of alleles; Ne effective number of alleles ;I, shannon's information index; Ho, observed heterozygosity; He, expected heterozygosity)

| plot | SD_SW | SD_SI | SD_J | S | Sample | Na | Ne | I | Ho | He |
| --- | --- | --- | --- | --- | --- | --- | --- | --- | --- | --- |
| D0101 | 2.539 | 0.892 | 0.821 | 22 | 22 | 6.447 | 3.903 | 1.372 | 0.532 | 0.634 |
| D0104 | 1.496 | 0.639 | 0.650 | 10 | 21 | 6.184 | 3.768 | 1.355 | 0.517 | 0.632 |
| D0108 | 2.790 | 0.907 | 0.829 | 29 | 21 | 6.684 | 4.062 | 1.408 | 0.536 | 0.642 |
| D0211 | 2.171 | 0.797 | 0.645 | 29 | 48 | 7.790 | 4.134 | 1.436 | 0.527 | 0.639 |
| D0307 | 2.528 | 0.864 | 0.723 | 33 | 35 | 7.658 | 4.018 | 1.426 | 0.530 | 0.633 |
| D0403 | 2.234 | 0.831 | 0.734 | 21 | 34 | 7.237 | 3.924 | 1.412 | 0.541 | 0.637 |
| D0411 | 2.643 | 0.875 | 0.743 | 35 | 39 | 7.290 | 4.064 | 1.411 | 0.533 | 0.635 |
| D0601 | 2.568 | 0.895 | 0.798 | 25 | 21 | 6.237 | 3.818 | 1.340 | 0.536 | 0.626 |
| D0606 | 1.937 | 0.712 | 0.610 | 24 | 47 | 8.026 | 4.189 | 1.451 | 0.533 | 0.642 |
| D0609 | 2.838 | 0.914 | 0.792 | 36 | 24 | 7.026 | 4.043 | 1.416 | 0.534 | 0.643 |
| D0613 | 2.867 | 0.921 | 0.820 | 33 | 16 | 5.263 | 3.354 | 1.230 | 0.531 | 0.599 |
| D0704 | 2.274 | 0.843 | 0.715 | 24 | 24 | 6.737 | 4.016 | 1.383 | 0.544 | 0.636 |
| D0711 | 2.626 | 0.891 | 0.797 | 27 | 26 | 6.395 | 3.680 | 1.341 | 0.536 | 0.620 |
| D0808 | 2.784 | 0.911 | 0.811 | 31 | 25 | 6.395 | 3.899 | 1.355 | 0.538 | 0.623 |
| D0902 | 2.229 | 0.825 | 0.662 | 29 | 18 | 6.132 | 3.857 | 1.360 | 0.535 | 0.634 |
| D0904 | 2.216 | 0.841 | 0.707 | 23 | 25 | 6.395 | 4.157 | 1.394 | 0.554 | 0.644 |
| D1006 | 2.571 | 0.842 | 0.729 | 34 | 15 | 5.842 | 3.768 | 1.337 | 0.539 | 0.630 |
| D1013 | 2.472 | 0.830 | 0.695 | 35 | 25 | 6.105 | 3.746 | 1.328 | 0.521 | 0.618 |
| D1109 | 1.979 | 0.784 | 0.640 | 22 | 23 | 5.711 | 3.471 | 1.259 | 0.504 | 0.600 |
| D1201 | 2.622 | 0.893 | 0.805 | 26 | 21 | 5.763 | 3.496 | 1.272 | 0.546 | 0.611 |
| D1205 | 2.566 | 0.887 | 0.770 | 28 | 38 | 7.026 | 3.815 | 1.391 | 0.555 | 0.637 |
| D1211 | 2.802 | 0.904 | 0.824 | 30 | 9 | 4.500 | 3.144 | 1.138 | 0.522 | 0.570 |
| D1309 | 2.782 | 0.902 | 0.782 | 35 | 18 | 6.132 | 3.987 | 1.350 | 0.558 | 0.631 |
| D1313 | 2.890 | 0.916 | 0.834 | 32 | 5 | 3.395 | 2.648 | 0.987 | 0.568 | 0.547 |

Table S5 Plot information (ELE, mean elevation; CON, convexity; SLO, slope).

| Plot | Group | ELE/m | CON | SLO | Plot | Group | ELE/m | CON | SLO |
| --- | --- | --- | --- | --- | --- | --- | --- | --- | --- |
| D0101 | Ⅲ | 496.62 | −9.70 | 9.70 | **D0711** | Ⅰ | 455.90 | −5.65 | 28.38 |
| D0104 | Ⅳ | 501.90 | −5.05 | 12.25 | **D0808** | Ⅱ | 495.70 | −2.82 | 37.83 |
| D0108 | Ⅲ | 488.76 | −6.81 | 29.57 | **D0902** | Ⅰ | 530.51 | −0.26 | 20.98 |
| D0211 | Ⅰ | 499.13 | 4.92 | 28.14 | **D0904** | Ⅳ | 548.41 | 4.90 | 18.66 |
| D0307 | Ⅰ | 493.79 | −13.80 | 7.79 | **D1006** | Ⅰ | 530.58 | 7.21 | 40.55 |
| D0403 | Ⅳ | 530.86 | 1.10 | 31.41 | **D1013** | Ⅰ | 442.46 | −4.05 | 26.47 |
| D0411 | Ⅱ | 481.81 | −2.24 | 26.85 | **D1109** | Ⅳ | 490.87 | −3.51 | 47.04 |
| D0601 | Ⅲ | 535.28 | −0.89 | 15.97 | **D1201** | Ⅲ | 512.58 | 17.44 | 21.66 |
| D0606 | Ⅲ | 528.53 | 1.01 | 32.38 | **D1205** | Ⅳ | 548.62 | 29.74 | 27.63 |
| D0609 | Ⅱ | 493.97 | 1.97 | 32.56 | **D1211** | Ⅲ | 464.58 | −78.82 | 31.20 |
| D0613 | Ⅱ | 446.21 | −3.24 | 9.88 | **D1309** | Ⅱ | 494.19 | 28.36 | 26.32 |
| D0704 | Ⅳ | 548.03 | 3.03 | 36.12 | **D1313** | Ⅱ | 456.42 | −41.86 | 25.77 |

Table S6 Soil properties (Mean ± SE (n =3) SOC, Soil organic carbon; TN, total nitrogen; TP, total phosphate; TK, total potassium).

| Plot | SOC g/kg | | TN g/kg | TP g/kg | | TK g/kg | | pH | | C:N g/kg | |
| --- | --- | --- | --- | --- | --- | --- | --- | --- | --- | --- | --- |
| D0101 | 19.08 ± 0.18 | | 3.74 ± 0.02 | 0.33 ± 0.003 | | 20.40 ± 0.01 | | 5.21 ± 0.06 | | 5.10 ± 0.03 | |
| D0104 | 48.45 ± 0.73 | | 2.51 ± 0.02 | 0.25 ± 0.001 | | 18.63 ± 0.12 | | 4.75 ± 0.07 | | 19.34 ± 0.04 | |
| D0108 | 46.36 ± 0.39 | | 3.18 ± 0.01 | 0.31 ± 0.01 | | 20.57 ± 0.18 | | 4.74 ± 0.11 | | 14.57 ± 0.06 | |
| D0211 | 51.92 ± 0.52 | | 2.30 ± 0.01 | 0.18 ± 0.01 | | 20.93 ± 0.13 | | 5.03 ± 0.08 | | 22.62 ± 0.04 | |
| D0307 | 103.85 ± 1.28 | | 3.77 ± 0.01 | 0.31 ± 0.01 | | 20.40 ± 0.23 | | 4.91 ± 0.13 | | 27.54 ± 0.08 | |
| D0403 | 74.31 ± 0.53 | | 3.05 ± 0.03 | 0.27 ± 0.01 | | 20.20 ± 0.15 | | 4.86 ± 0.09 | | 24.39 ± 0.05 | |
| D0411 | 96.13 ± 1.10 | | 5.09 ± 0.03 | 0.31 ± 0.01 | | 19.23 ± 0.12 | | 5.00 ± 0.07 | | 18.88 ± 0.04 | |
| D0601 | 74.74 ± 1.25 | | 4.87 ± 0.02 | 0.43 ± 0.01 | | 21.33 ± 0.18 | | 4.62 ± 0.10 | | 15.35 ± 0.06 | |
| D0606 | 53.02 ± 0.53 | | 2.72 ± 0.02 | 0.24 ± 0.01 | | 22.60 ± 0.15 | | 4.77 ± 0.09 | | 19.48 ± 0.05 | |
| D0609 | 67.77 ± 0.87 | | 3.83 ± 0.02 | 0.40 ± 0.01 | | 22.50 ± 0.15 | | 4.84 ± 0.09 | | 17.70 ± 0.05 | |
| D0613 | 42.04 ± 1.06 | | 3.14 ± 0.02 | 0.33 ± 0.01 | | 20.60 ± 0.12 | | 4.68 ± 0.07 | | 13.40 ± 0.05 | |
| D0704 | 134.13 ± 2.55 | | 5.14 ± 0.03 | 0.41 ± 0.003 | | 23.00 ± 0.06 | | 4.70 ± 0.09 | | 26.11 ± 0.02 | |
| D0711 | 90.90 ± 0.50 | | 4.05 ± 0.01 | 0.40 ± 0.01 | | 23.10 ± 0.18 | | 4.74 ± 0.07 | | 22.46 ± 0.06 | |
| D0808 | 73.39 ± 0.87 | | 4.23 ± 0.02 | 0.35 ± 0.003 | | 22.20 ± 0.15 | | 4.72 ± 0.09 | | 17.35 ± 0.05 | |
| D0902 | 54.84 ± 0.58 | | 4.56 ± 0.02 | 0.34 ± 0.01 | | 21.77 ± 0.13 | | 5.34 ± 0.08 | | 12.03 ± 0.04 | |
| D0904 | 105.75 ± 1.15 | | 4.45 ± 0.01 | 0.32 ± 0.01 | | 22.60 ± 0.15 | | 4.89 ± 0.09 | | 23.77 ± 0.05 | |
| D1006 | 122.82 ± 1.25 | | 4.44 ± 0.01 | 0.36 ± 0.01 | | 21.83 ± 0.15 | | 4.90 ± 0.09 | | 27.67 ± 0.05 | |
| D1013 | 52.49 ± 0.77 | | 3.58 ± 0.02 | 0.36 ± 0.01 | | 23.10 ± 0.10 | | 5.05 ± 0.06 | | 14.68 ± 0.03 | |
| D1109 | 90.05 ± 0.92 | | 3.99 ± 0.02 | 0.35 ± 0.01 | | 22.00 ± 0.15 | | 5.35 ± 0.09 | | 22.60 ± 0.05 | |
| D1201 | 102.10 ± 1.24 | | 4.83 ± 0.02 | 0.42 ± 0.01 | | 21.00 ± 0.10 | | 4.95 ± 0.06 | | 21.14 ± 0.03 | |
| D1205 | 66.49 ± 0.90 | | 3.17 ± 0.02 | 0.27 ± 0.004 | | 23.83 ± 0.14 | | 4.99 ± 0.08 | | 20.98 ± 0.05 | |
| D1211 | 50.13 ± 0.97 | | 3.77 ± 0.03 | 0.36 ± 0.003 | | 23.30 ± 0.17 | | 4.94 ± 0.10 | | 13.30 ± 0.06 | |
| D1309 | 72.47 ± 0.84 | | 3.37 ± 0.02 | 0.32 ± 0.01 | | 20.37 ± 0.13 | | 5.10 ± 0.08 | | 21.53 ± 0.04 | |
| D1313 | 52.74 ± 0.39 | | 2.98 ± 0.01 | 0.43 ± 0.01 | | 26.63 ± 0.18 | | 4.61 ± 0.10 | | 17.72 ± 0.06 | |
| Mean ± SD | | 72.7 ± 27.6 | 3.78 ± 0.801 | | 0.335 ± 0.06 | | 21.8 ± 1.69 | | 4.90 ± 0.21 | | 19.2 ± 5.32 |


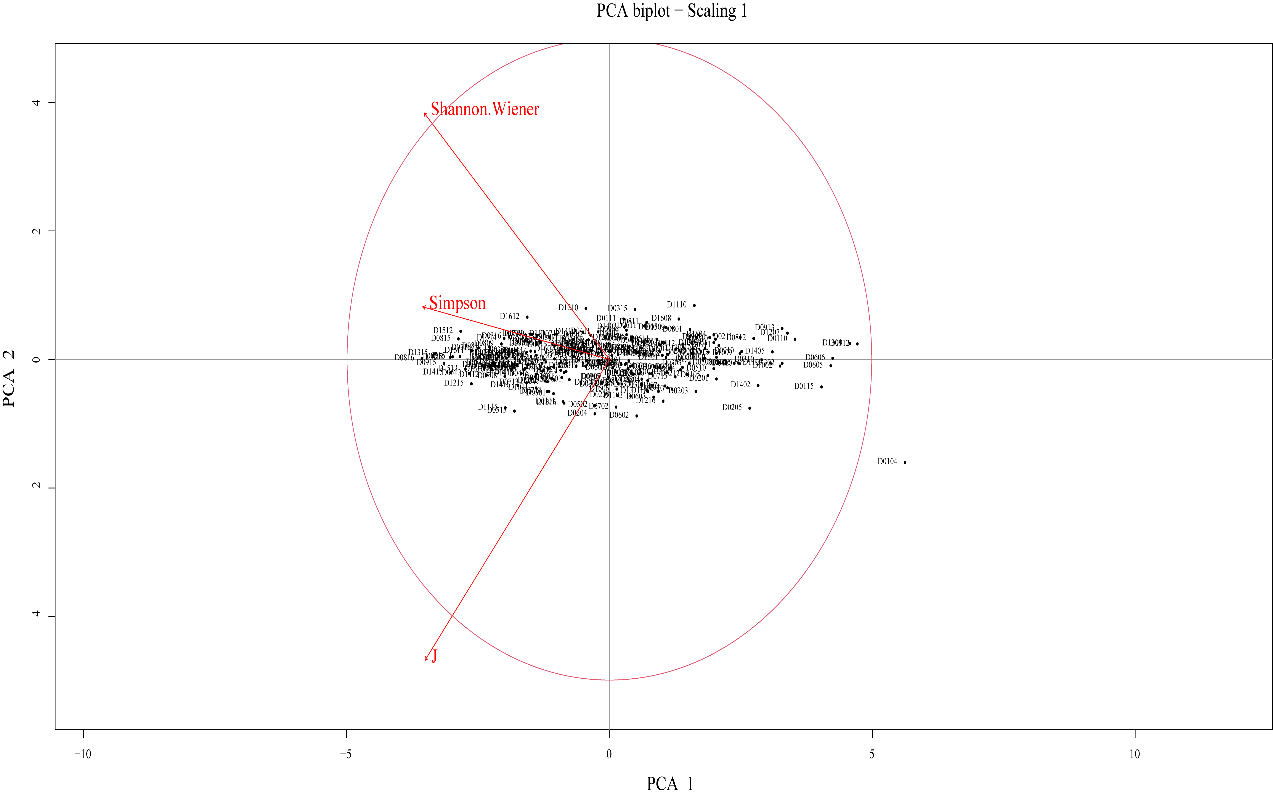


FIGURES1 Principal component analysis divides 256 samples into four components


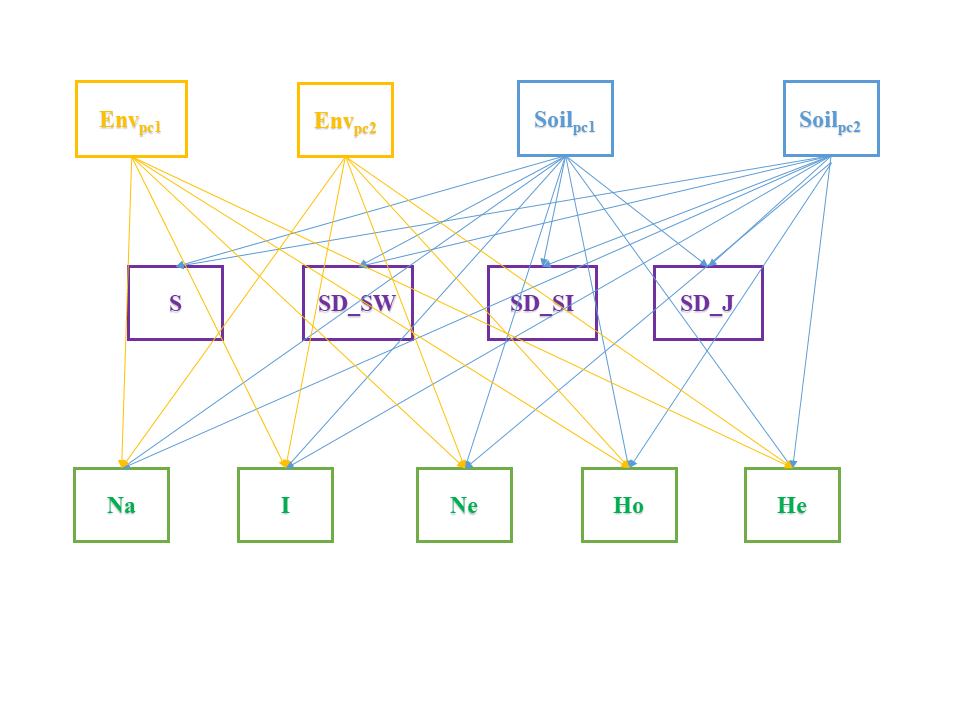
FIGURES2 Meta model for SEM

Reference

Fu, D. D., J. Wang, Y. F. Liu, and H. W. Huang. 2010. Isolation of Microsatellite Markers for Castanopsis fargesii ( Fagaceae). Journal of Tropical and Subtropical Botany **18**:541-546.

Huang, G. M., L. Hong, W. H. Ye, H. Shen, H. L. Cao, and W. Xiao. 2009. Isolation and characterization of polymorphic microsatellite loci in Castanopsis chinensis Hance (Fagaceae). Conservation Genetics **10**:1069-1071.

Jiang, Y., Z. H. Li, J. Y. Zhu, and H. L. Liu. 2015. Development and characterization of polymorphic microsatellite markers for Castanopsis hystrix (Fagaceae). Genetics and Molecular Research **14**:2436-2439.

Shi, M. M., S. G. Michalski, X. Y. Chen, and W. Durka. 2011a. Isolation by Elevation: Genetic Structure at Neutral and Putatively Non-Neutral Loci in a Dominant Tree of Subtropical Forests, Castanopsis eyrei. Plos One **6**.

Shi, Y. S., J. Zhang, K. Jiang, M. Y. Cui, and Y. Y. Li. 2011b. Development and Characterization of Polymorphic Microsatellite Markers in Castanopsis Sclerophylla (Fagaceae). American Journal of Botany **98**:E19-E21.

Ueno, S., H. Yoshimaru, T. Kawahara, and S. Yamamoto. 2003. A further six microsatellite markers for Castanopsis cuspidata var. sieboldii Nakai. Conservation Genetics **4**:813-815.
